# The Prevalence of Infectious Diseases Among Seventh-Day Adventists: A Systematic Review

**DOI:** 10.1177/15598276251370238

**Published:** 2025-08-20

**Authors:** Robert Krisztian Janko, Irmgard Haussmann, Ashok Patel

**Affiliations:** 1 Department of Health Sciences, 1725 Birmingham City University, Birmingham, UK (RKJ, IH, AP)

**Keywords:** infections, lung infection, nutritional deficiency

## Abstract

**Background:** Several studies have investigated the incidence of chronic diseases among Adventists, but less is known about the association between the prevalence of infectious diseases and the Adventist lifestyle. **Methods:** This systematic review adhered to the Preferred Reporting Items for Systematic Reviews and Meta-Analyses (PRISMA) guidelines and has been registered on PROSPERO under CRD42024502363. Relevant studies were searched in databases such as PubMed, Google Scholar, and Scopus. Observational studies reporting on the prevalence or occurrence of infectious diseases within the Adventist community were included if they were published in English language. The included studies were synthesised in the form of a narrative synthesis. **Results:** The outcomes related to infectious conditions identified were *Toxoplasma gondii*, *Helicobacter pylori*, antibodies to *Vibrio* species and Norwalk virus, upper respiratory infections (URIs), COVID-19 disease, H1N1 influenza, and infectious disease-related mortality. A lower prevalence of some infectious diseases was shown among Adventists. A study noted lower Toxoplasma gondii seroprevalence in Adventists, and another associated high fruit, vegetable, and water intake with fewer respiratory infections. **Conclusions:** The Adventist lifestyle may be associated with a lower prevalence of infectious diseases, likely due in part to the Adventist diet. However, further research is needed to clarify the relative contributions of individual lifestyle factors to these protective effects.

‘The patients received plant-based meals and none of the infected patients died or developed pneumonia’.

## Introduction

The Seventh-day Adventist church is a Protestant Christian denomination, mostly known for its promotion of a healthy lifestyle. Adventists are encouraged to eat a diet low in animal products, and high in fruits, vegetables, whole-grains, pulses, nuts and seeds, which is in strong agreement with evidence-based dietary guidelines such as those published by the World Health Organization (2020).^1^

Seventh-day Adventists have been the subjects of a number of epidemiological studies that have assessed the prevalence or incidence of chronic diseases such as cardiovascular disease, type 2 diabetes, and cancer. Most notable is the Adventist Health Study-2,^2^ which recruited over 96,000 Adventists, and compared mortality rates between vegetarians and non-vegetarians. The results showed that vegetarian Adventists had a significantly lower risk of mortality than meat eating Adventists. Other studies have shown that Adventists experienced lower cancer^3^ and ischaemic heart disease^4^ risk.

The Adventist lifestyle extends beyond diet. Adventists adhere to 8 health ‘laws’, which they believe are vital for good health. These health principles focus on the consumption of a healthy diet, the observance of the Sabbath as a day of rest, the importance of clean air, physical exercise, the healing properties of sunlight and the consumption of water as one’s main fluid source, and the importance of faith in God and temperance in all things.^5^ Adherence to these principles has clearly proven to be an effective strategy to prevent chronic diseases, however, less is known about the potential association between the Adventist lifestyle and the prevalence or incidence of infectious diseases. Given that many aspects of the Adventist lifestyle, such as nutrition, have been shown to play a role in immune function and the treatment and prevention of infectious diseases,^6^ there is compelling rationale for investigating the potential association between the Adventist lifestyle and the prevalence of infectious diseases.

Therefore, this study aimed to synthesise the available evidence regarding the prevalence of infectious diseases among Seventh-day Adventists.

## Methods

This systematic review has been conducted in line with the Preferred Reporting Items for Systematic Review and Meta-Analyses (PRISMA) guideline and has been registered on PROSPERO under CRD42024502363.

## Ethics

No ethical approval was required for this systematic review from the Health, Education and Life Sciences Faculty Academic Ethics Committee of Birmingham City University, UK as no participants were involved in this study since it was a systematic review of already existing literature.

## Inclusion and Exclusion Criteria

Peer-reviewed studies published in English language were searched in PubMed, Google Scholar and Scopus. They were included in this systematic review if they recruited Adventist participants and reported on the prevalence or incidence of infectious diseases. Studies that had mixed populations were excluded unless they reported distinguishable data for Adventists. Studies performed at Adventist hospitals were generally excluded, unless they specifically reported data for patients who were Adventist.

## Database Search

The above-mentioned databases were searched using the following search strategy:

(Adventist OR Seventh Day Adventist OR SDA) AND (infection OR infectious OR ‘infectious disease’ OR infected OR virus OR bacteria OR ‘bacterial infection’ OR ‘viral infection’).

The titles and abstracts of articles were initially screened after retrieval from the databases. Those that were deemed relevant were read in full and the articles that met the inclusion criteria were included in this systematic review.

## Data Extraction

The process of data extraction was completed by RKJ and reviewed by AP and IH using a standardised data extraction form. Bibliographic information, study characteristics data, participant demographics, the presence of a control group, infectious disease outcomes, funding source and information concerning statistical analysis were extracted. The quality and potential bias in the included studies were evaluated using the Newcastle-Ottawa Scale (NOS),^7^ which uses a scoring system between 0 to 9, where the higher scores represent a lower risk of bias. A modified version of the NOS was used for cross-sectional study designs validated by Modesti et al. (2016).^8^

The NOS evaluates the quality of studies based on the domains of selection, which assesses how well the study selected and defined the exposed and non-exposed groups, measured exposure, and ensured outcomes were not present at baseline if relevant, comparability, which examines whether the study controlled for confounding variables, and outcome, which evaluates the method of outcome assessment, the length of follow-up, and the adequacy of follow-up.

## Narrative Synthesis

Studies were grouped based on geographical location, the type of infectious disease investigated and study design in line with the Synthesis Without Meta-analysis (SWiM) guidelines.^24^ The studies’ results were summarised in a table format as well as descriptively, highlighting the incidence or prevalence rate of the infectious disease investigated. Where possible, comparisons were made across studies investigating the same outcome. However, significant heterogeneity in the investigated outcomes prevented a statistical summary of the results.

## Results

**Figure 1.**
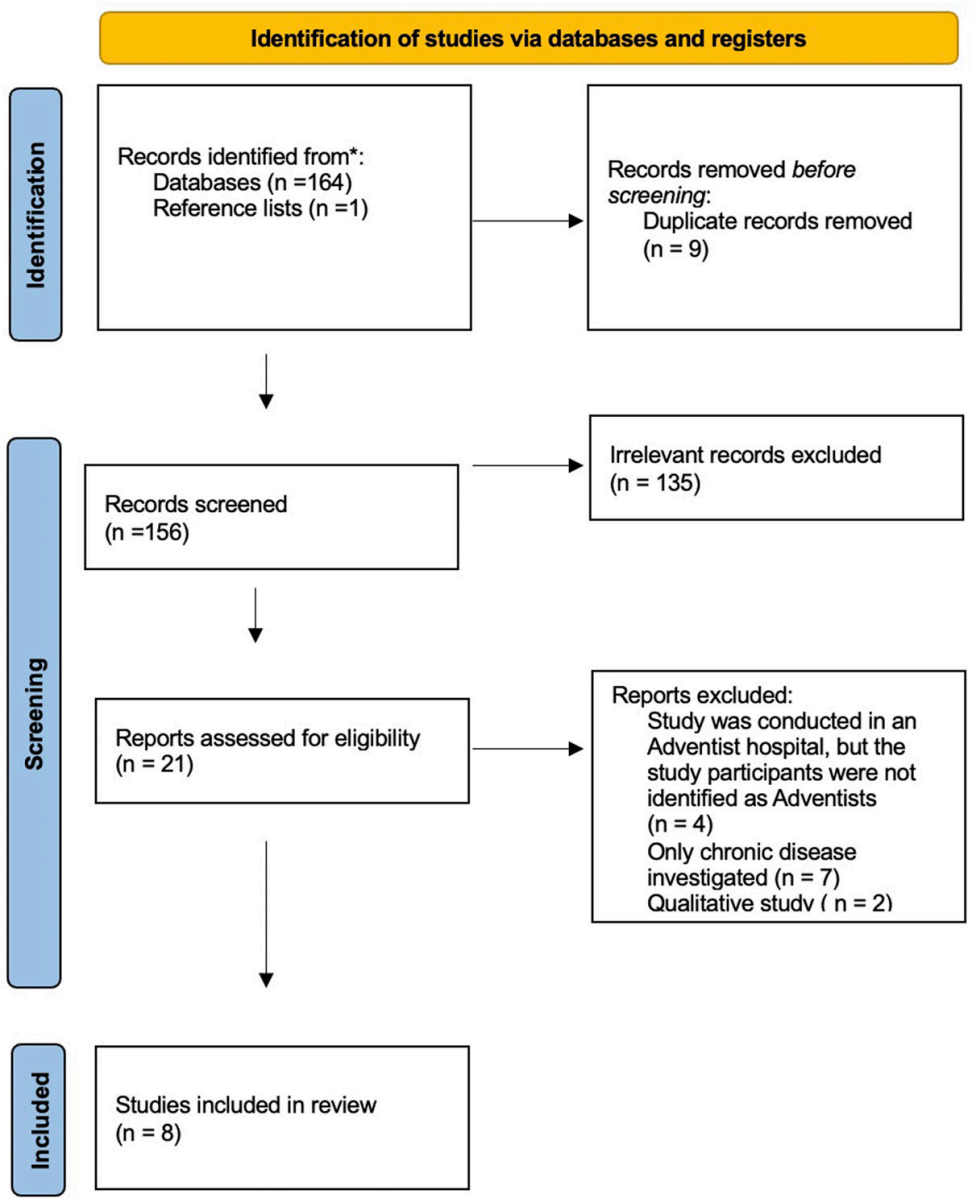
PRISMA flow diagram of study screening and selection.

One hundred and sixty-five articles were retrieved after the initial search of the literature. Of these, 21 were read in full and eight have been included in this systematic review (Figure 1). The characteristics of the included studies and their main results are presented in Table 1.

**Table 1.** Results and Characteristics of Included Studies.

Author(s)/year of Publication	Location/Setting	Study Design	Sample Size	Adventist Lifestyle Characteristics (if Mentioned)	Infectious Disease(s) Investigated	Prevalence / Incidence Rates	Key Findings (Related to Adventist Lifestyle)
Jacobs 1956^10^	United States of America	Cross-sectional seroprevalence survey	46	Vegetarian diet with no meat	T. gondii	21.6% among Adventists	No control groups.
Hopkins 1990^11^	United States of America	Cross-sectional study	262	Vegetarian diet abstaining from alcohol, caffeine, and meat	*Helicobacter pylori* infection	Overall seropositivity rate was 35% in both Adventists and controls.	No correlation found with dietary habits, Adventist status, sex, geographic location, prior military service, or travel history
Lefkowitz 1992^12^	United States of America	Cross-sectional seroprevalence survey	258	Abstinence from shellfish, smoking, and alcohol; adherence to a lacto-ovo-vegetarian diet.	Antibodies to Vibrio species and Norwalk virus	Not specified	No significant association between shellfish consumption or contact with shellfish and antibody response to *Vibrio cholerae* O1 or Norwalk virus.
Roghmann 1999^13^	United States of America	Cross-sectional seroprevalence study	251	Adherence to a lacto-ovo-vegetarian diet containing no meat, seafood, or poultry.	Toxoplasma gondii infection	Odds Ratio (OR) for T. gondii infection among Adventists compared to controls: 0.21, 95% CI: 0.09-0.46, *P* < 0.01	Seventh-day Adventists had a significantly lower risk of T. gondii infection compared with the controls.
Liao 2006^14^	United States of America	Cross-sectional analysis of a sample of the AHS-2 cohort	41,725	Not specified	Upper respiratory infections (URI)	Inverse relationship between intake of fresh fruits, vegetables, water, and odds of a URI	Higher intake of fruits and vegetables and regular water consumption is associated with a decreased risk of URI. OR for 7+ servings of fruits (compared to 1 or less) = 0.75 (95% CI: 0.69-0.82) OR for 7+ servings (compared to less than 2) of vegetables = 0.80 (95% CI: 0.75-0.85)
Büssing 2022^15^	Germany	Cross-sectional survey	1494	None	COVID-19	19% of participants reported having had the infection	No control groups.
Kahleova 2022^16^	United States of America	Cross-sectional	120	Plant-based diet including grains, fruits, nuts, and vegetables.	H1N1 influenza	No deaths or pneumonia among 90 infected Adventist faculty staff and students	Treatment included rest in bed and patients were asked to apply warm soaks to the throat and chest and were provided with a plant-based diet.
Abris et al, 2024^17^	United States of America	Longitudinal cohort study of the AHS-2 sample.	12,515	Vegetarian	Infectious disease mortality	HR for vegetarians of mortality due to infectious diseases was 0.57 (95% CI: 0.40, 0.82) as compared with non-vegetarian Adventists.	Lower infectious disease mortality observed among vegetarians.

Seven of the included studies were conducted in the United States with one study each from Germany and Indonesia. Six of the included studies used a cross-sectional design, whilst one was a commentary that included a report of H1N1 infection rates from a Seventh-day Adventist seminary.^16^ The studies published by Widiani and Djula (2022)^18^ and Riedel and Trecartin, 2024^19^ were excluded as it was a qualitative research study, which did not report data on COVID-19 risk.

## COVID-19 Infection

One of the included studies reported COVID-19 infection prevalence to be as low as 19% among the recruited Adventists, however the authors did not conduct any subgroup analysis relating to the prevalence of COVID-19 infection based on various lifestyle characteristics such as diet.^15^ Furthermore, the study did not recruit any non-Adventists as controls.

## Toxoplasma gondii

Two of the included studies investigated Toxoplasma gondii (T. gondii) infections. Roghmann et al (1999)^13^ investigated the seroprevalence of T. gondii among Adventists and non-Adventists living in Maryland, US. The researchers hypothesised that Adventists would have a lower T. gondii infection prevalence as a large portion of Adventists do not consume meat and it was anticipated that meat exposure would increase disease risk. The study recruited 251 participants in total, 105 of whom were Adventists and 146 non-Adventists. Controls were local fishermen, seafood processing plant workers, and visitors to a seafood festival, and both groups were required to undergo a blood draw to allow for the determination of blood antibody levels against T. gondii. Of the 250 samples analysed, 31% had evidence of a previous T. gondii infection. The study showed that the seroprevalence of T. gondii was significantly lower among Adventists (25%) as compared with the control group (50%) across all age groups (OR 0.21, 95% confidence interval 0.09-0.46, *P* < .01). Overall, this study concluded that being an Adventist was associated with a significantly lower risk of T. gondii infection. The results of the other T. gondii seroprevalence survey published by Leon Jacobs^10^ in 1957 found a somewhat higher seroprevalence (21.6%) among vegetarian Seventh-day Adventists, however this earlier study had a smaller sample size (n = 46) and did not have a control group.

The certainty of evidence for the study published by Roghmann et al (1999)^13^ can be considered moderate due to the robust statistical analysis and narrow confidence intervals. In contrast, the evidence certainty for Jacobs (1956)^10^ is low because of the absence of a control group and the lack of reported confidence intervals.

### Norwalk virus, *Vibrio cholerae* and Vibrio Vulnific Seroprevalence

Another serosurvey included in this systematic review was published by Lefkowitz et al (1992),^12^ which investigated the prevalence of antibodies against the shellfish associated pathogens *Norwalk virus*, *Vibrio cholerae* and *Vibrio vulnific*. Participants were shellfish workers (n = 140) and Seventh-day Adventists (n = 127) who served as the low-contact or low-consumption group. Of the shellfish workers, 83.7% reported occupational contact with raw shellfish at least once a week, as opposed to only 6% in the Adventist group. The study did not find a significant difference in the seroprevalence of *V. vibrio or the Norwalk virus* between the two groups, however shellfish workers were found to have a higher seroprevalence of the unencapsulated phase variant of *V. vulnificus* compared with the Adventist group. Seropositivity was highest for the Norwalk virus in the overall sample at 70%. The certainty of evidence for *Norwalk virus*, *Vibrio cholerae*, and *Vibrio vulnificus* seropositivity is low due to the absence of confidence intervals and insufficient statistical power to detect small differences in seroprevalence.

### Helicobacter pylori

The third serosurvey included was published by Hopkins et al (1990)^11^ and it compared the seroprevalence of *Helicobacter pylori* among Seventh-day Adventists (n = 94) and two groups of non-Adventists (n = 168). The sampling method of participants is not reported; therefore, it is likely that the study used convenience sampling. The recruited Adventists were selected from six congregations on the eastern shore of Maryland, with one of the control groups geographically matched from rural and small-town areas, whilst the other based in the Baltimore-Washington, DC, area (urban). The results showed no difference in the seroprevalence of *H. pylori* between the Adventists (35%) and the controls (35%), even after controlling for age in the analysis. On the other hand, age was a significant determined of seropositivity in the whole sample. The results constitute a moderate level of certainty due to the lack of confidence intervals presented and lack of randomisation used.

## Upper Respiratory Tract Infections

The study with the largest sample size recruited 41 725 participants, all of whom were part of the AHS-2 cohort.^14^ The authors of this study analysed the risk of upper respiratory tract infections (URI) within a 12-month period and found that there was a 25% reduction in the odds of having a URI (OR 0.75, 95% CI: 0.69-0.82) among those eating seven or more servings of fresh fruits compared with those consuming them once a day or less. Similarly, eating seven or more servings of vegetables was associated with a 20% lower risk of URIs (OR 0.80, 95% CI: 0.75-0.85) compared with eating two or less servings per day. Importantly, the associations remained unchanged in the fully adjusted models. The authors concluded that a diet rich in fresh fruits, vegetables and water decreased the risk of URIs. The large sample size, precise effect estimates as reflected by the narrow confidence intervals, and robust statistical adjustments support the certainty of the findings.

## The Rest of the Included Studies

A commentary published by Kahleova and Barnard (2022)^16^ has also been included in this systematic review as it presents data relating to a Seventh-day Adventist seminary in Minnesota from 90 H1N1 influenza pandemic (1918) infected patients. The patients received plant-based meals and none of the infected patients died or developed pneumonia. The article reports that mortality at the time was 6.7% among the US Army. Only one of the included studies reported COVID-19 infection prevalence data, however the authors did not conduct any subgroup analysis relating to the prevalence of COVID-19 infection based on various lifestyle characteristics such as diet, and the study did not recruit non-Adventists as controls.^15^ Finally, the latest publication of the AHS-2 cohort by Abris et al^17^ included data on infectious disease mortality in Adventists aged 65 and 85, stratified by dietary status. Vegetarians in the 65-year age group had a significantly lower mortality rate due to infectious diseases than non-vegetarians, however, the study did not specify which infectious diseases accounted for those deaths. Finally, the latest publication of the AHS-2 cohort included data on infectious disease mortality in Adventists aged 65 and 85, stratified by dietary status. Vegetarians in the 65-year age group had a significantly lower mortality rate due to infectious diseases than non-vegetarians, however, the study did not specify the exact infectious diseases.

The risk of bias assessment utilising the Newcastle-Ottawa Scale (NOS) is presented in Table 2, which indicates a variance in the quality and risk of bias in the included studies. The articles published by Lefkowitz et al (1992)^12^ and Abris et al (2024)^17^ appear to have the lowest risk of bias with scores of 8 and 7 respectively. In contrast, Kahleova and Barnard (2022)^16^ scored the lowest with only 1 star each, indicating a higher risk of bias, particularly due to the low scores obtained in the selection and comparability domains.

**Table 2.** Risk of Bias Assessment.

Study ID	Selection (max 5 Stars)	Comparability (max 2 Stars)	Outcome/Exposure (max 3 Stars)	Total (max 10 Stars)
Roghmann 1999^13^	1	2	2	5
Liao 2006^14^	2	2	2	6
Lefkowitz 1992^12^	3	2	3	8
Kahleova 2022^16^	0	0	1	1
Hopkins 1990^11^	2	1	2	5
Jacobs 1957^10^	1	0	1	2
Büssing 2022^15^	1	1	1	3
Abris 2024^17^	3	2	2	7

## Discussion

This was the first systematic review that investigated the prevalence of infectious diseases among Seventh-day Adventists. Due to the presence of significant heterogeneity and the low number of studies focussing on the same infectious disease quantitative analysis in the form of a meta-analysis was not possible.

Two of the included studies assessed the prevalence of T. gondii infection among Seventh-day Adventists, but only one of them had a control group.^13^ In this study, the seroprevalence of T. gondii was significantly lower than in the control group (OR 0.21, 95% CI 0.09-0.46, P .0001). The authors’ conclusion highlights the potentially protective effect of meat abstinence against T. gondii, which is confirmed by a meta-analysis^20^ which showed that the consumption of raw and undercooked meat and fish greatly increases the risk of T. gondii infection. Interestingly, the meta-analysis did not find a significant association between the consumption of unpasteurised milk or raw eggs and the seroprevalence of T. gondii, which may explain how vegetarian Adventists in the included studies, who presumably consume dairy products and eggs, had a lower risk of infection. Household exposure to raw meat and the duration of time on a vegetarian diet among the Adventists were not assessed in the included studies, both of which are important factors that could have influenced the results. Furthermore, Roghmann et al (1999)^13^ showed that the seroprevalence of T. gondii was highest among those over the age of 60 in both groups, which agrees with the results of a recently published study^21^ of non-Adventists, which reported that age was a significant risk factor for T. gondii exposure.

The only study^15^ which focused on COVID-19 disease did not recruit a control group, nor did it conduct a subgroup analysis based on lifestyle factors. This is relevant since the lower intake of animal products and the consumption of plant-based diets have been associated with lower COVID-19 incidence rates^22^ and a lower risk of severe COVID-19 infection.^23^ However, the Adventist lifestyle does not just recommend a vegetarian or plant-based diet, it also advises against alcohol consumption and smoking, both of which have been associated with a lower prevalence of COVID-19 disease and a lower severity of infection.^9,24^

The study^14^ with the largest sample size showed that the high consumption of fruits, vegetables and water was associated with a lower risk of URI. This finding has been confirmed by later studies,^25^ which reported an association between fruit and vegetable intake and a lower URI incidence.

## Strengths and Limitations

One of the main limitations of this systematic review is that most studies were cross-sectional in nature, which does not allow for the assessment of disease incidence rates.

Many of the included studies relied on self-reported data concerning dietary habits, whilst one of the studies did not even assess dietary intake, it just assumed the Adventists were all non-meat-eaters.^11^

Furthermore, most of the included studies utilised convenience sampling instead of random sampling which could have led to selection bias. Further to this, controls in some of the studies may not be representative of the general population. For instance, the studies published by Roghmann et al (1999)^13^ and Lefkowitz et al (1992)^12^ recruited local fisherman and seafood processing plant workers as controls, however, they are at an increased exposure to the investigated pathogens and may not serve as suitable comparators to Adventists.

Studies investigating T. gondii infection^10,13^ did not assess household exposure to raw meat, which may have led to an underestimation of the true effect of the exposure on the outcome.

On the other hand, this was the first attempt to systematically review the literature to synthesise the available evidence concerning the Adventist lifestyle and its potential association with infectious diseases, which provides a broad perspective on the topic and highlights the need for further studies. The synthesis process adhered to systematic principles outlined in the SWiM guidelines^26^ to ensure transparency and reproducibility.

No conclusive findings can be drawn from this systematic review due to the large heterogeneity between the studies owing to the different diseases investigated and their high risk of bias arising mostly from the lack of observational period inherent to cross-sectional study designs, which makes it difficult to establish the temporality and directionality of the relationship between the Adventist diet and lifestyle and infectious diseases. Additionally, this systematic review identified further issues, including the use of inappropriate or absent control groups and a lack of rigour in statistical analyses. Future research should incorporate subgroup analyses based on the various aspects of the Adventist lifestyle and include a duration of follow-up in their designs to allow for the investigation of infectious disease incidence rates.

## Conclusions

This systematic review suggests a potential association between the Adventist lifestyle and a lower prevalence of certain infectious diseases, with the diet appearing to play the most prominent role. However, the contribution of other lifestyle factors remains unclear, and the low certainty of evidence limits the ability to draw firm conclusions.

Importantly, this systematic review also serves to identify the clear lack of robust research in this area. While conclusive findings cannot be drawn, the observed trends provide a rationale for future well-conducted prospective studies that can evaluate specific lifestyle factors among Adventist populations in relation to infectious disease outcomes.
